# A Multi-Pronged Computational Pipeline for Prioritizing Drug Target Strategies for Latent Tuberculosis

**DOI:** 10.3389/fchem.2020.593497

**Published:** 2020-12-14

**Authors:** Ushashi Banerjee, Santhosh Sankar, Amit Singh, Nagasuma Chandra

**Affiliations:** ^1^Department of Biochemistry, Indian Institute of Science, Bangalore, India; ^2^Center for Infectious Disease Research, Indian Institute of Science, Bangalore, India; ^3^Center for Biosystems Science and Engineering, Indian Institute of Science, Bangalore, India

**Keywords:** latent tuberculosis, target identification, systems biology, chemoinformatics, lead identification, metabolic modeling, response network

## Abstract

Tuberculosis is one of the deadliest infectious diseases worldwide and the prevalence of latent tuberculosis acts as a huge roadblock in the global effort to eradicate tuberculosis. Most of the currently available anti-tubercular drugs act against the actively replicating form of *Mycobacterium tuberculosis* (*Mtb*), and are not effective against the non-replicating dormant form present in latent tuberculosis. With about 30% of the global population harboring latent tuberculosis and the requirement for prolonged treatment duration with the available drugs in such cases, the rate of adherence and successful completion of therapy is low. This necessitates the discovery of new drugs effective against latent tuberculosis. In this work, we have employed a combination of bioinformatics and chemoinformatics approaches to identify potential targets and lead candidates against latent tuberculosis. Our pipeline adopts transcriptome-integrated metabolic flux analysis combined with an analysis of a transcriptome-integrated protein-protein interaction network to identify perturbations in dormant *Mtb* which leads to a shortlist of 6 potential drug targets. We perform a further selection of the candidate targets and identify potential leads for 3 targets using a range of bioinformatics methods including structural modeling, binding site association and ligand fingerprint similarities. Put together, we identify potential new strategies for targeting latent tuberculosis, new candidate drug targets as well as important lead clues for drug design.

## 1. Introduction

Tuberculosis, despite being one of the oldest infectious diseases known to humankind with a history dating back to 70,000 years, continues to affect millions of people, causing over 0.1 million deaths globally every year (Barberis et al., 2017; WHO, 2018). A major roadblock in TB eradication is the capacity of the causative organism *Mycobacterium tuberculosis* (*Mtb*) to remain in a non-replicative state inside an infected person for decades without causing any symptoms. An approximate 1.7 billion of the world's population is thought to harbor latent tuberculosis (Ai et al., 2016; WHO, 2018). These individuals carry about a 10% lifetime risk of developing active TB, a risk that increases in the presence of factors such as malnutrition or immunocompromised conditions. The treatment of latent TB, therefore, becomes an important requirement for successful control and management of TB around the world.

Clinically, latent TB signifies a state of persistent immune response to *M.tuberculosis* infection, where the host remains asymptomatic and the bacilli are in a non-replicating dormant state but have the potential to reactivate when the host gets immunocompromised. *Mtb* owes its ability to reside for long periods inside the host in a dormant condition to its capacity of rewiring its own metabolism to a viable yet non-replicative state which allows its maintenance and evasion of host immune mechanisms (Gengenbacher et al., 2010). It gets engulfed by alveolar macrophages at the site of infection, but prevents phagosomal maturation and thus stops its own subsequent destruction by the host immune system, forming granuloma (Russell, 2001; Pethe et al., 2004). In most of the cases, the granuloma becomes solid and fibrous, successfully containing the infection inside and establishing latent TB infection (Gengenbacher and Kaufmann, 2012). In order to survive inside the host cells, the bacteria must adapt to their hostile environment where they are challenged with various stresses such as low nutrients, hypoxia, low pH etc. (Schnappinger et al., 2003). Multiple *in vitro* models have elucidated different aspects of the biochemical and molecular correlates of *Mtb* dormancy. A metabolic downshift induced by the granuloma environment is a predominant change that the pathogen goes through in order to achieve the dormant state (Betts et al., 2002). The glyoxylate shunt has been proven to be essential for bacterial survival during dormancy (McKinney et al., 2000; Gengenbacher et al., 2010). Hypoxia has been one of the most leveraged stress conditions in induction of dormancy *in vitro* and it has led to the identification of the Dos regulon that controls about 50 genes and regulates bacterial adaptation into non-replicative state in hypoxia (Park et al., 2003; Voskuil et al., 2004). Utilization of lipids as an alternative carbon source has also been suggested as an important adaptation in dormancy (Santucci et al., 2016).

Most of the anti-tubercular drugs target cellular processes in actively replicating cells such as the transcription, cell wall synthesis and the generation of ATP (Campbell et al., 2001; Timmins and Deretic, 2006; Nagabushan and Roopadevi, 2014). These drugs lose their potency against latent tuberculosis due to the metabolic alterations seen in the dormant bacteria. At present, for lack of better options, the existing anti-tubercular drugs are administered for prolonged periods to latent TB individuals. Although, both monotherapy and combination therapy have been reported to have similar efficacy, the longer duration monotherapy has been in common practice due to the possibility of drug interaction between rifampicin/ rifapentine and other drugs like the ones used for treatment of HIV (Fox et al., 2017; Saha et al., 2020). This prolonged antibiotic course is often perceived as unnecessary for an asymptomatic condition by both the patient and the clinician, leading to lack of adherence and an average of 20–30% treatment completion rate in most cases (Horsburgh, 2004). Therefore to successfully eradicate TB when about 2 billion of the world's population could be acting as the pathogen reservoir, new shorter drug regimens are urgently required.

Although drug discovery for tuberculosis has gained momentum with the emergence of MDR and XDR *Mtb* strains, the number of new drugs has not increased significantly. An ideal new TB drug should lead to a shorter treatment duration than those in practice, have minimal toxicity and interaction with other drugs and kill different subpopulations observed in clinical TB with different replication rates (Lechartier et al., 2014). Metronidazole, pretomanid and certain hydrazone molecules have shown promise against non-replicating *Mtb* (Wayne and Sramek, 1994; Stover et al., 2000; Bonnett et al., 2018). High-throughput compound screening and bioinformatics analysis have also identified potential drug candidates against dormant tuberculosis (Cho et al., 2007; Raman et al., 2008; Defelipe et al., 2016). Yet most of the proposed drugs in different phases of clinical trial target processes like DNA replication and cell wall synthesis which have limited efficacy against dormant bacteria (Dietze et al., 2008; Gler et al., 2012; Naidoo et al., 2017). Since the complete sequencing of *Mtb* genome (Cole et al., 1998), different omics-based studies have provided insights into the biology of tuberculosis in active and latent conditions and have been utilized for drug target identification (Lew et al., 2011; Goff et al., 2020). Although a drug-to-target pipeline has been traditionally more successful in discovering new TB drugs than a target-to-drug pipeline, it has not been able to identify potential drugs for specifically killing the non-replicating pathogen (Lechartier et al., 2014). On the other hand, drugs like Bedaquiline, that target the ATP homeostasis are shown to have high efficacy in killing dormant bacteria as they target processes necessary for bacterial survival in dormancy (Koul et al., 2008; Rao et al., 2008; Kaushik et al., 2019). It shows that in the case of latent TB, a target-based drug approach might be more efficient in identifying new drugs targeted toward processes crucial for bacterial dormancy.

In this work, we have used a systems biology based computational pipeline to strategically identify six different potential drug targets for tackling latent TB. We further select three of these as candidate targets and use multiple bioinformatics and chemoinformatics approaches to identify lead clues for these targets that may aid in the process of drug discovery. Our approach utilizes a combination of transcriptome analysis, genome-scale metabolic modeling, condition-specific genome-wide protein-protein interaction networks and bioinformatics based on protein sequences, protein structure, binding sites and chemoinformatics that analyse the chemical structures and topologies of the ligand molecules.

## 2. Methods

### 2.1. Curation of Publicly Available Transcriptome Data

The objective of this study was to identify potential drug targets for latent TB. Transcriptome data was not publicly available from dormant pathogens residing in infected host tissues. But, a significant number of studies have been successful in establishing dormancy of *Mtb in vitro* with the application of different stress conditions, which partially replicates the multitude of stresses faced by the pathogen inside the host granuloma environment. We searched in the public repository Gene Expression Omnibus (GEO) (Edgar et al., 2002) for transcriptome data of dormant *Mtb* and selected the datasets listed in Table 1 based on the following criteria: (a) presence of well-defined timepoints of *Mtb* dormancy, i.e., establishment of bacteriostasis and non-culturability induced by external stress, (b) samples from exponential/ logarithmic growth phase in absence of an external stress, (c) data generated with microarray or RNAseq techniques, (d) ≥ 3 samples per condition and time points, from which we selected one dataset each (that has data for the maximum number of time points) representing different stress conditions.

**Table 1:** Transcriptome datasets from GEO database considered in this study for *in vitro Mtb* for 4 different dormancy models.

**GEO ID and platform**	**Dormancy inducing condition**	**Conditions and number of samples**	**Stages and timepoints of dormancy**	**References**
GSE8786 (GPL5714)	Hypoxia	Exponential growth (35), pre-dormancy (6) and Non-replicative persistence (29)	NRP1 (Day 6–8), NRP2 (Day 10–20), NRP3 (Day 30–80)	Voskuil et al., 2004
GSE10391 (GPL4388)	Multiple stress (Low O2, acidic pH, low glycerol)	Exponential growth (47), pre-dormancy (39) and dormancy (8)	Dormancy (Day 9–18)	Deb et al., 2009
GSE66408 (GPL18768)	Potassium Deficiency	Exponential growth (3) and dormancy (9)	Early (Day 14), Mid (Day 24) and Late dormancy (Day 34)	Ignatov et al., 2015
GSE84554 (GPL4057)	Iron Deficiency	Exponential growth (24), pre-dormancy (12) and dormancy (18)	Dormancy (Day 7–14)	Kurthkoti et al., 2017

### 2.2. Gene Expression Analysis

Raw data from the selected datasets listed in Table 1 were downloaded from GEO. Each dataset was pre-processed and normalized separately using EdgeR and limma package from Bioconductor in R statistical environment (Robinson et al., 2010; Huber et al., 2015; Ritchie et al., 2015). In brief, the two-channel microarray datasets were background corrected, followed by within and between array normalization with loess and quantile methods, respectively. The RNAseq dataset, GSE66408, was subjected to the TMM scaling normalization method (Robinson and Oshlack, 2010). Normalized gene expression values were used for differential gene expression calculation between dormancy and exponential growth conditions with moderated t-statistics and Benjamini–Hochberg's method to control for false discovery rate (FDR). Genes with ≥ 1.5-fold change in expression, with FDR ≤ 0.05 were considered to be statistically significant differentially expressed genes (DEGs).

### 2.3. Contextualization of *Mtb* Genome-Scale Metabolic Model and Analysis of Reaction Fluxes

Genome-scale metabolic models (GEM) are large-scale *in silico* reconstructions of metabolic networks of an organism that describes the association between genes, proteins and reactions. We selected the most updated GEM of *Mtb*, iEK1011, which combined the previous GEMs and is manually curated for recent literature, for this study (Kavvas et al., 2018). This model contains 1228 metabolic reactions, 998 metabolites and 1011 genes.

Flux balance analysis (FBA) is a constraint-based modeling approach that analyzes the flow of metabolites through a metabolic network, i.e., fluxes of metabolic reactions. The principle and procedure for FBA have been thoroughly described elsewhere (Raman and Chandra, 2009; Orth et al., 2010). To identify the metabolic changes occurring in *Mtb* when it enters a dormant state, we integrated gene expression data from different growth states into the metabolic model iEK1011, using the algorithm E-Flux (Colijn et al., 2009). E-Flux utilizes continuous gene expression data to set the upper and lower bounds of the reaction fluxes using gene-protein-reaction (GPR) rule from the model. Therefore, a reaction associated with highly expressed genes in a given condition will allow higher flux through it while maximizing the defined objective function. We have used E-Flux (Colijn et al., 2009) to contextualize the iEK1011 model by integrating the corresponding transcriptome data. Next, we have limited the exchange reactions of the respective stress condition to replicate the experimental environment. For example, in hypoxia-induced dormancy model, the oxygen exchange reaction, that allows entry of oxygen into the cell, is limited to reproduce the hypoxia state. We then computed the fluxes through all the reactions for each growth and dormancy condition and time point with FBA using the biomass objective. The reactions with no flux or flux <1E-06 in both exponential and dormancy conditions were removed from further study. A reaction was considered to be significantly perturbed in dormancy if flux fold-change ≥ 1.5, as compared to corresponding exponential phase flux. The analysis was performed using COBRA toolbox in MATLAB R2018b (Becker et al., 2007; Heirendt et al., 2019).

### 2.4. Updation of Knowledge-Based *Mtb* Protein-Protein Interaction Network (MtbPPIN)

A high-confidence knowledge-based protein-protein interaction network, MtbPPIN, was updated from the previously constructed in-house *Mtb* network (Ghosh et al., 2013) using information from databases as well as primary literature. First, all interaction information available in the database STRING (Szklarczyk et al., 2015) for *Mtb* H37Rv strain was downloaded. Each interaction in the database is associated with a confidence score on a scale of 0 to 1000, where a higher score corresponds to higher confidence interactions. Only the interactions with a confidence score ≥ 800 were retained for MtbPPIN. Next, functional interactions were manually curated from KEGG database (Kanehisa and Goto, 2000; Kanehisa, 2019; Kanehisa et al., 2019) *Mtb* pathways. Further physical associations were added to the network from the high-throughput screening studies (Wang et al., 2010; Wu et al., 2017). High confident transcriptional regulatory interactions were added to the network from multiple primary studies (Balázsi et al., 2008; Zeng et al., 2012; Galagan et al., 2013; Minch et al., 2015). Only the interactions between protein-coding genes were retained. Directionality information was incorporated into the network wherever available. Interactions representing transcriptional regulation were directed from regulator to target, signaling or metabolic interactions were directed from upstream to downstream of the pathways. Interactions signifying physical binding were considered as bidirectional. Unknown functionality interactions were undirected. After a final round of manual curation, the high-confidence genome-scale network, MtbPPIN consists of 3,980 nodes (proteins) and 53,208 edges (interactions) with 62% directionality. About 96% of the known *Mtb* H37Rv proteome is included in MtbPPIN. MtbPPIN interactions are provided in Supplementary File 1.

### 2.5. Response Network Analysis

Response network analysis is an in-house computational pipeline that integrates transcriptome data in protein-protein interaction networks to capture the important perturbations in a cell or organism in a certain condition (Sambarey et al., 2013, 2017; Padiadpu et al., 2016; Bhosle et al., 2020). We generated response networks for each dormancy stage of each condition by weighing the nodes and edges with transcriptome information using the following Equations (1), (2), and (3). The feasibility of the flow of information through the nodes can be calculated as the path cost through those nodes (Equation 4).

Since the weights of the edges are inversely proportional to the node weights, the lower the path cost the higher the flow of information. The paths with the least cost capture the highest perturbations in a given condition. The top 0.001 percentile paths from both the active and repressed response networks were used to obtain the top perturbed response networks of dormancy stages (Dormancy_TPN_). For cases where transcriptome data from multiple timepoints are available for one dormancy stage, a union of the Dormancy_TPN_ of the corresponding timepoints were considered. All networks and pathways were visualized using Cytoscape 3.7 (Shannon et al., 2003).

### 2.6. Structural Studies of Shortlisted *Mtb* Proteins

For the shortlisted candidate target molecules, we studied their protein sequences, structures and binding sites using well-established bioinformatics methods. The input sequences of all regulated proteins were retrieved from the Mycobrowser in FASTA format (Kapopoulou et al., 2011). The structure template appropriate for query sequence was identified using the popular fold recognition software called MUSTER (Wu and Zhang, 2008). MUSTER is based on an extension of the traditional sequence profile-profile alignment algorithms (PPA) with additional structural features such as prediction of secondary structure elements, solvent accessibility, backbone dependent dihedral angle and hydrophobic scoring matrix. In addition to sequence derived information, used by other programs, new features incorporated by MUSTER aid in deriving structural relationships thereby increasing the sensitivity of template being chosen. The quality of template that is aligned with the query sequence was verified based on the Z-score cutoff scheme as reported in the original MUSTER paper. Z-score is obtained either by dividing raw alignment score (R_score_) by full length alignment (L_full_) or by dividing R_score_ by partial length alignment (L_partial_). Z-score value greater than 7.5 indicates a significant alignment of query sequence over template structures. Finally, an all-atom structure reconstruction was built using MODELER, a software for comparative protein structure modeling (Webb and Sali, 2016). The quality and robustness of models were investigated using ERRAT (Colovos and Yeates, 1993).

### 2.7. Consensus Binding Site Detection Pipeline

Numerous site prediction algorithms are available online each exploiting distinct ways to find binding pockets in the proteins. Consensus identification of sites from multiple methods yields high confidence binding sites essential for this work. Three independent pocket detection methods used for this study were (i) PocketDepth, a geometry-based method (Kalidas and Chandra, 2008), (ii) SiteHound, energy-based (Ghersi and Sanchez, 2009) and (iii) FPocket, based on Voronoi tessellations (Le Guilloux et al., 2009). PocketDepth, an inhouse method, uses the notion of a depth factor to identify putative binding pockets for a given protein structure. It is a geometry-based method which encompasses the protein as a volume of grid cells each of size 1Å. All grid cells are then classified either as internal (if the cell is within 2Å from atom occupied grids) or external (everything else). A depth-first graph traversal search along all six faces of the cube gives a list of connected cells defining the pockets. The second algorithm, SiteHound, which is an energy-based method, predicts a site by finding energetically favorable regions on the protein surface using Molecular Interaction Fields (MIF). Lastly, FPocket relies on Voronoi tessellation by constructing a set of small alpha spheres in proteins that connect four atoms on its boundary and all connected atoms are equidistant to the alpha sphere center. All three methods run on different principles and hence served as independent methods from which only those sites predicted by all three methods were considered as a consensus pocket. The minimum residue overlap of 4 residues was considered for consensus pocket identification and by default pocket definition from FPocket was used for further analysis.

### 2.8. Multiple Approaches of Associating Targets With Lead Molecules

Three methods were used for associating ligand molecules to the selected targets, based on (i) binding site sub-structures, (ii) whole protein structures level and (iii) protein sequences. The site-based association uses the structural similarity between sites as a basis to transfer function from one protein to another. It broadly comprises the following steps—comparing binding sites against known ligand sites from Protein Data Bank (PDB) (Berman et al., 2000) and transferring ligands, measuring similarity between PDB ligands and drugs in DrugBank (Wishart et al., 2008), and associating drugs to the query protein. On the contrary, the second approach uses whole fold similarity for making a ligand association transfer. For each drug entry available in the DrugBank database, UniProt Ids of protein to which it gets recognized were taken. Following that, the primary sequence data of all drug binding proteins were downloaded from the UniProt database (UniProt Consortium, 2019). A large-scale protein structural modeling exercise was then set up using the MUSTER program. Structural templates suitable for modeling each sequence were taken based on Z-score 7.5. Proteins whose Z-score value is less than 7.5 were not considered further in this work as they do not have a reliable template for predicting their structures. Finally, sequence-based annotation involves identification of putative drug-like molecules based on similarity with sequences in the ChEMBL database (Gaulton et al., 2012). The binding energy of each drug molecule with the target proteins was calculated using AutoDock (Forli et al., 2016).

## 3. Results

### 3.1. Identification of Potential Drug Targets for Latent TB With Systems-Level Analysis

**Figure 1:**
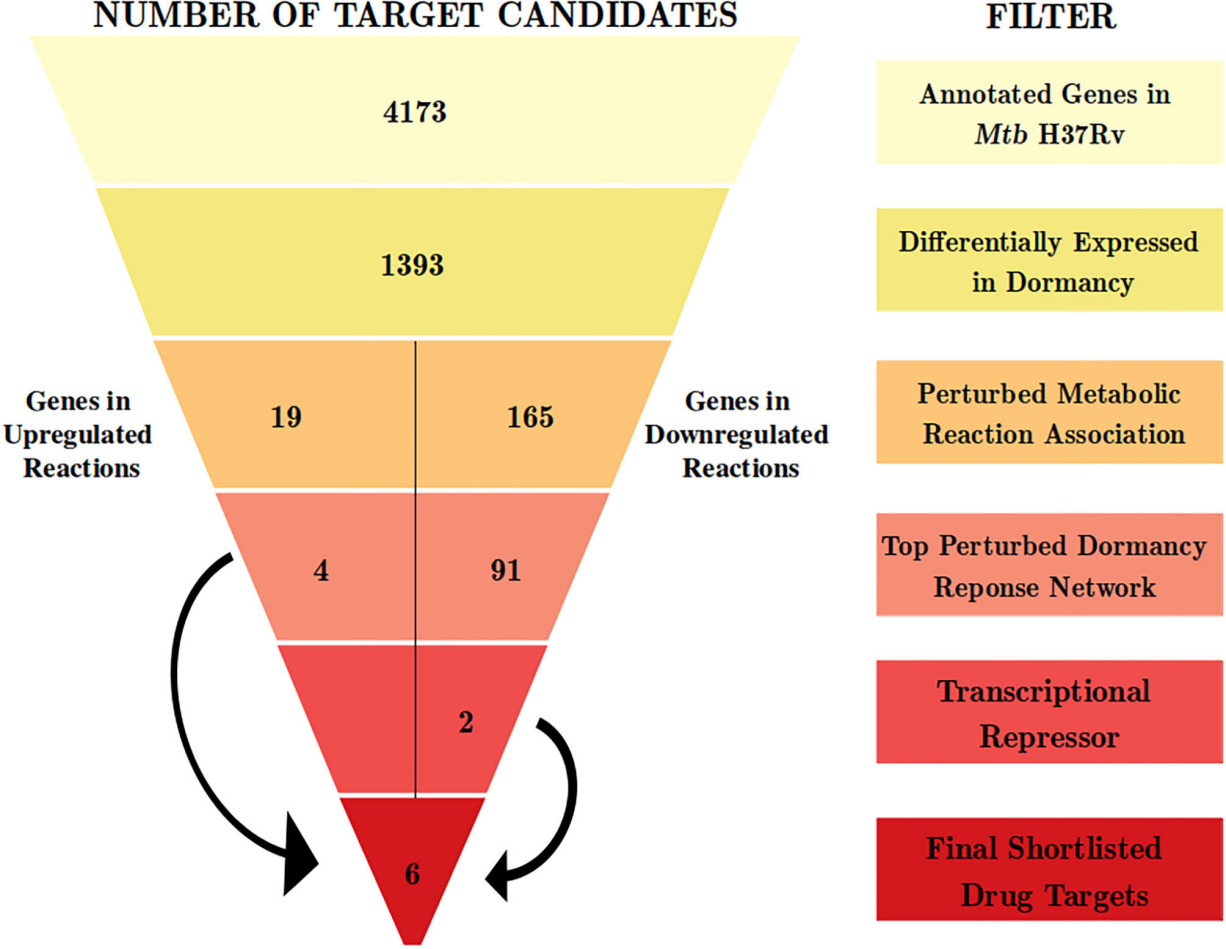
The computational pipeline used for drug target candidate identification for latent tuberculosis. The pipeline involved stepwise filters based on gene expression, metabolic modeling, and protein-protein interaction network analysis to shortlist proteins crucial for *Mtb* dormancy. Six proteins were shortlisted as potential drug target candidates from this pipeline.

With an aim of identifying targetable cellular mechanisms that have increased activity in non-dividing *Mtb*, we configure a multi-step computational pipeline that performs genome-scale metabolic modeling followed by a contextualized genome-wide protein-protein interaction network analysis (Figure 1). Different steps in the pipeline serve as filters to select only those genes that satisfy the criteria defined at that step. The filters correspond to gene expression variations or alterations in metabolic fluxes in associated reactions, as described below.

#### 3.1.1. Genome-Wide Profiling of Alterations in *Mtb* During Dormancy in Different Models Identify Most Likely Perturbations Inside Latent Host Granuloma

**Figure 2:**
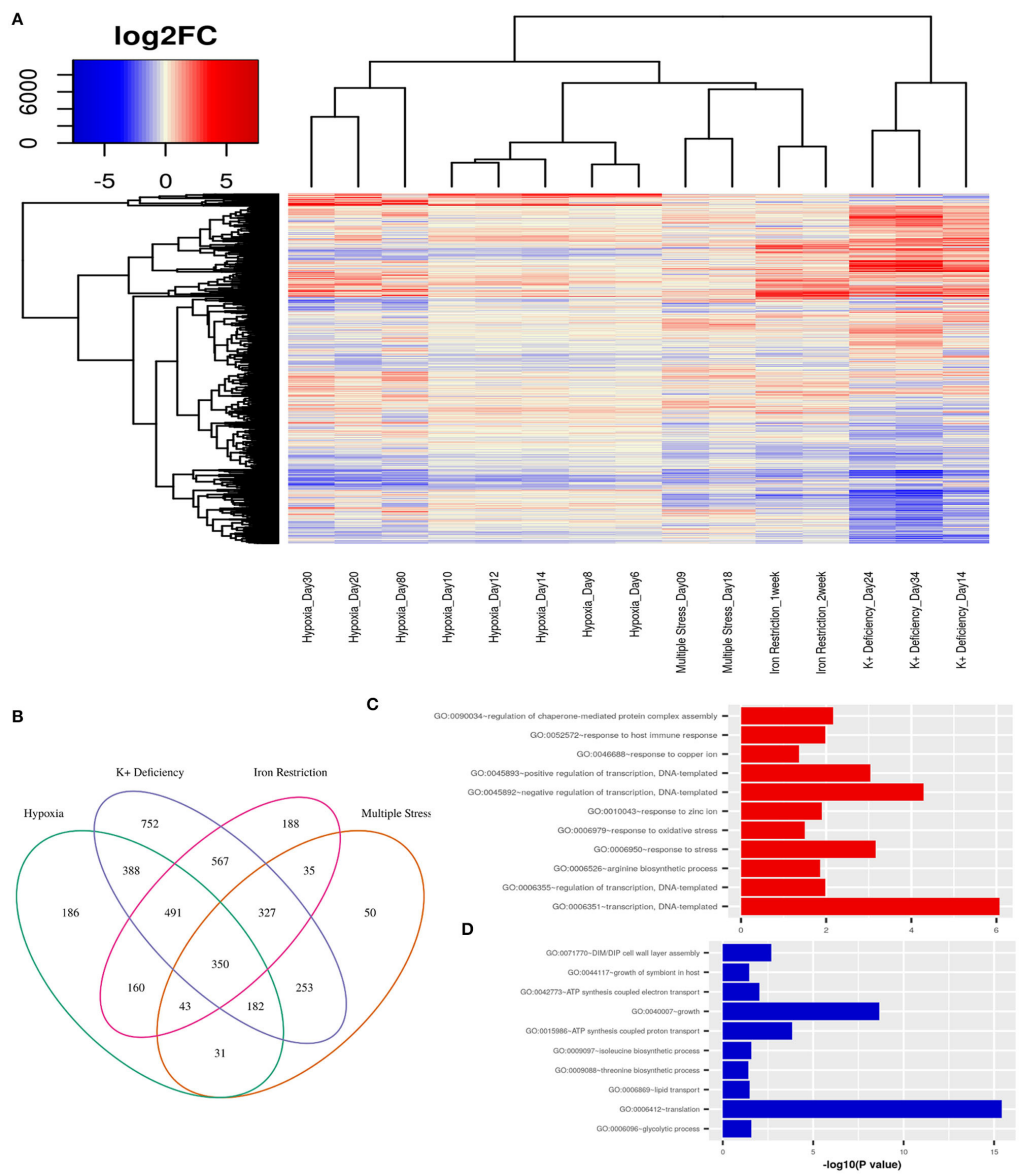
**(A)** Variations in gene expression profiles of *Mtb* in different *in-vitro* dormancy models with respect to actively replicating *Mtb*. Gene expression alterations are depicted as log2(fold changes) over an exponential growth condition taken as the reference in each dataset. Red signifies upregulation and blue signifies downregulation of gene expression. Each column in the heatmap shows the gene expression in the time point and model indicated in the figure. **(B)** A Venn diagram showing the overlap between DEGs (fold change ≥ ±1.5, FDR ≤ 0.05) between different dormancy models w.r.t. exponential growth. 350 genes were differentially regulated in all four dormancy models at some time point and 1393 genes were differentially regulated in at least 3 models. **(C)** GO: biological processes overrepresented in the commonly upregulated genes amongst dormancy conditions and **(D)** GO: biological processes overrepresented amongst the genes commonly downreguated across multiple dormancy conditions as analyzed using the enrichment tool in DAVID database (Huang et al., 2009).

First, we obtain an unbiased view of the alterations in the dormant *Mtb* cells as compared to their actively replicating counterparts. We selected publicly available transcriptome data from various *in vitro* dormancy models of *Mtb* that employed different kinds of stress factors, as described in the section 2, to induce a non-replicating stage in the bacterial culture (Voskuil et al., 2004; Deb et al., 2009; Ignatov et al., 2015; Kurthkoti et al., 2017). We selected one dataset per condition, which included hypoxia, multiple stress, potassium deficiency and iron deficiency, to represent the different kinds of stresses faced by *Mtb* in the granuloma environment. In each of the dormancy inducing conditions, dormant cells showed a significant number of genes to be differentially regulated (FC≥ ±1.5, FDR ≤ 0.05) when compared to cells growing in exponential phase (Figure 2A). In the datasets where different stages of dormancy were classified (GSE8786 and GSE66408), the transcriptome showed a gradual change in the perturbation from early to later time points. Although a large number of genes were perturbed in each condition, the overall change in the transcriptome varied between different conditions as could be observed from the clustering pattern that clearly separates the dormancy conditions from each other (Figure 2A). Each of the stress conditions individually caused an up or downregulation in a large portion of the *Mtb* transcriptome, but only 350 genes were differentially regulated in all four of the conditions and 1393 genes were DEGs in any three of the conditions studied (Figure 2B). Interestingly, the commonly downregulated genes are enriched in protein synthesis, metabolism and growth, indicative of a non-replicative state whereas the commonly upregulated genes were enriched in different biological processes such as “response to host immune response,” “response to oxidative stress,” and “response to copper ion” which are indicative of responses similar to those observed in dormant *Mtb* surviving in latent TB granuloma (Figures 2C,D). This shows that although individual stress-inducing conditions trigger responses in *Mtb* that are mostly specific to that particular stress, the transcriptome perturbation that occurs in multiple conditions does indeed replicate the state of the pathogen inside latent host granuloma, where it stays metabolically dormant as a survival strategy against the host immune response.

We used the transcriptome analysis of dormancy conditions not only to ascertain the applicability of *in vitro* dormancy models in studying latent TB in the host, but also as our first step of the filter in the drug target identification pipeline for dormant *Mtb*. *Mtb* H37Rv has 3994 known proteins annotated in Uniprot (UniProt Consortium, 2019) and 4,173 genes annotated in Mycobrowser (Kapopoulou et al., 2011). The first filter applied in the drug target identification pipeline was that of a gene being a DEG in 3 or more of the *in vitro* dormancy conditions studied. This resulted in 1,393 genes from *Mtb* passing the first filter (Figure 1 and Supplementary File 2).

#### 3.1.2. Contextualized Genome-Scale Metabolic Modeling Elucidates Most Perturbed Metabolic Reactions in Dormant *Mtb*

**Figure 3:**
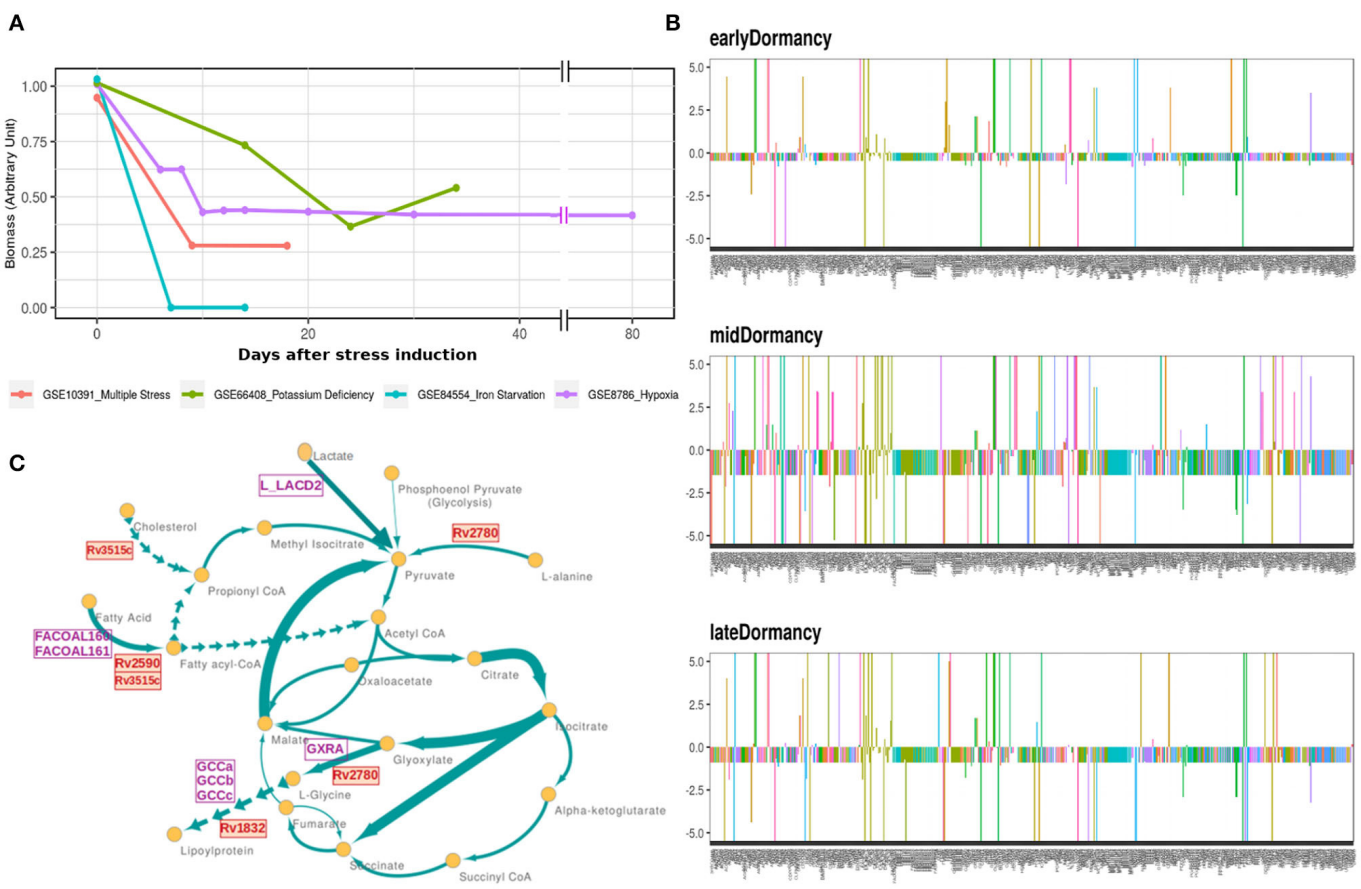
**(A)** Optimal flux through the hypothetical biomass reaction from iEK1011 in different timepoints of all the models showing a significant reduction in biomass generation in dormancy compared to exponential growth. **(B)** Flux fold change of all non-zero reactions in Potassium deficiency model of dormancy at 3 different stages in comparison to the exponential growth phase. X axis in each contains the reactions with non-zero fluxes and Y axis shows the fold change value. Most of the reactions are downregulated during dormancy. **(C)** A representative subgraph of metabolic pathways focused on the TCA-glyoxylate cycle of central carbon metabolism in dormant state *Mtb* in multiple stress induced model. The thickness of the edges is directly proportional to flux through the reactions. Yellow circles signify metabolites. Purple squares indicate reactions from the 14 commonly upregulated reactions across dormancy models. Red squares highlight the genes eventually identified as drug target candidate further down the pipeline.

*Mtb* goes through extensive metabolic remodeling to achieve the non-replicative yet viable state during latency (Boshoff and Barry, 2005; Schubert et al., 2015; Baker and Abramovitch, 2018). We utilized the most updated Genome Scale Metabolic Model of *Mtb*, iEK1011, integrated with gene expression data, to generate a contextualized metabolic model for each dormancy condition (as described in section 2) (Colijn et al., 2009; Kavvas et al., 2018). A consequent FBA on the exponential growth as well as dormant *Mtb* specific iEK1011 provided the fluxes through the reactions in the respective condition. Contextualization of the GEM with transcriptome data showed a 50-100% reduction in the generalized biomass production reaction in the dormancy conditions from the exponential growth phase (Figure 3A), showing the capability of the method to replicate the dormancy phenotype. Fluxes through 421 reactions were found to be altered in three or the more dormancy conditions (Supplementary Files 3, 4). Of these, 407 reactions were downregulated in all the four dormancy conditions compared to exponential growth phase whereas 14 of the reactions showed significant upregulation across dormancy condition (Figure 3B and Supplementary Figures 1A–C). The downregulated reactions belonged to different amino acid synthesis pathways, cell wall and mycolic acid biosynthesis, fatty acid metabolism, aerobic respiration etc. (Figure 3C and Supplementary File 5). Among the reactions with significant upregulation across dormancy models were key reactions in the fatty acid metabolism, glyoxylate cycle and glycine cleavage system (Figure 3C and Table 2).

**Table 2:** Reactions commonly upregulated in 3 or more dormancy models.

**Reaction ID**	**Reaction name**	**Subsystem**	**GPR**
ASP1DC	Aspartate 1-decarboxylase	Alanine, Aspartate, and Glutamate Metabolism	Rv3601c
P5CRx	Pyrroline-5-carboxylate reductase(nadh)	Arginine and Proline Metabolism	Rv0500
GCCc	Glycine-cleavage complex	Citric Acid Cycle	Rv3303c
EX_h2	H2 exchange	Extracellular exchange	
DESAT16	Palmitoyl CoA desaturase n C160CoA n C161CoA	Fatty acid metabolism	Rv3229c
FACOAL160	Fatty acid CoA ligase hexadecanoate	Fatty Acid Metabolism	Rv3826 or Rv1529 or Rv1185c or Rv2590
FACOAL161	Fatty-acid CoA ligase hexadecenoate	Fatty Acid Metabolism	Rv0035 or Rv0099 or Rv0119 or Rv0166 or 30 others
GCCa	Glycine-cleavage complex	Glycolysis/Gluconeogenesis	Rv1832
GCCb	Glycine-cleavage complex	Glycolysis/Gluconeogenesis	Rv2211c and Rv1826
GXRA	Glycine dehydrogenase (deamidating)	Glyoxylate metabolism	Rv2780
QRr	QRr	Oxidative phosphorylation	Rv3777
L_LACD2	L-Lactate dehydrogenase (ubiquinone)	Pyruvate metabolism	Rv1872c
FHL	Formate-hydrogen lyase	Redox metabolism	Rv0084
H2td	Hydrogen transport	Transport	

*Details of subsystems and GPRs (Gene Protein Rule) are taken from iEK1011*.

From this analysis, the differentially regulated reactions were selected for further steps in the target identification pipeline. The up and downregulated reactions without GPR information were filtered out, leaving 12 and 356 reactions into the pipeline, respectively. The 12 upregulated reactions were associated with 45 genes, whereas the 356 downregulated reactions were associated with 390 genes. Of these, 19 and 165 genes, respectively, overlapped with the list of 1393 DEGs from the previous step (Figure 1 and Supplementary File 2). These DEGs are involved in perturbed metabolic reactions in the dormant state of *Mtb* and were taken forward to the next step of the pipeline.

#### 3.1.3. Response Network Analysis Identifies Top-Ranked Hubs Associated With Perturbations in Dormant *Mtb*

**Figure 4:**
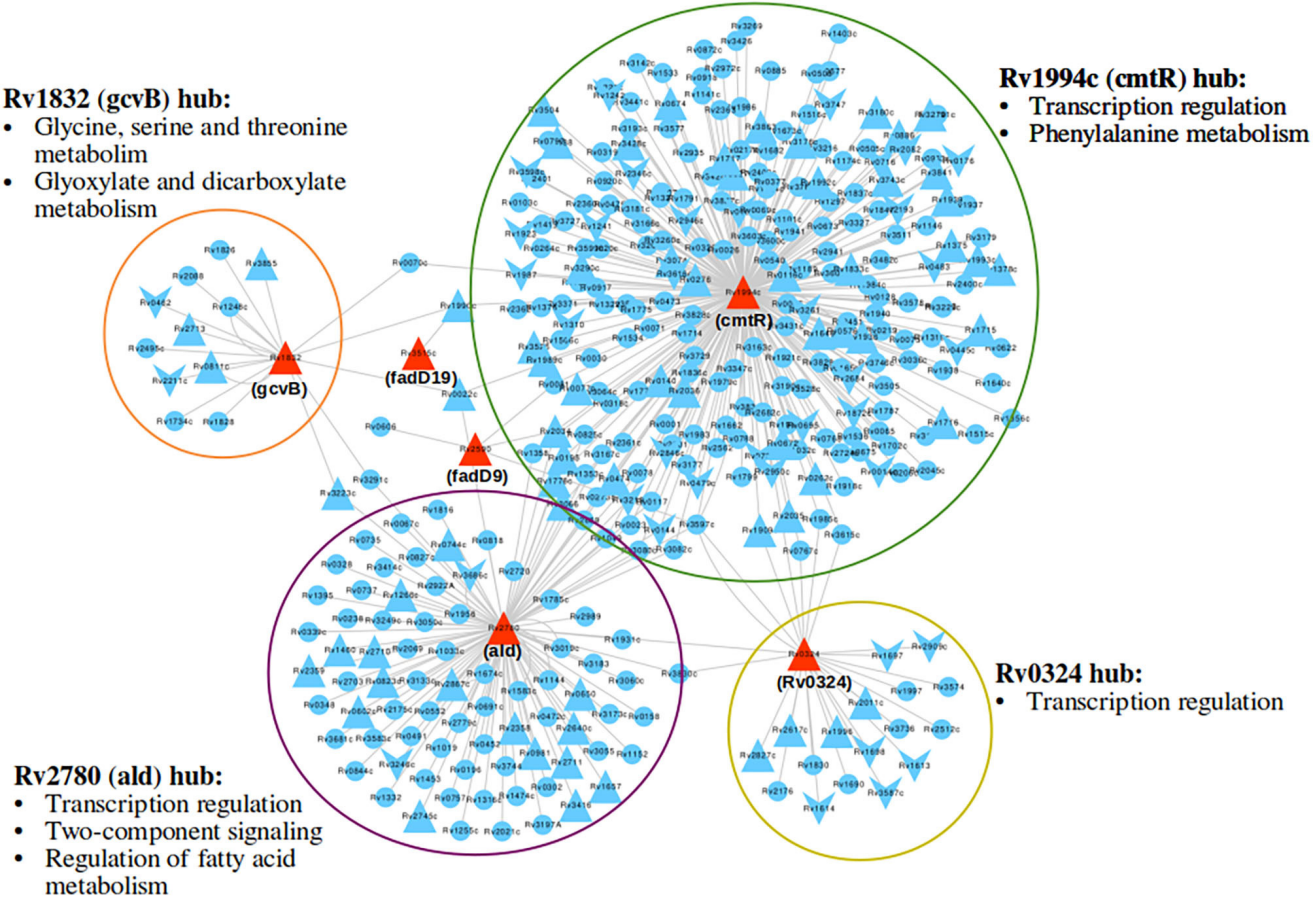
A subnetwork of union dormancy response network for the 6 shortlisted drug target candidates and their first neighbors. The target candidates are colored in red. Upward and downward triangular nodes signify up and downregulation of gene expression, respectively. cmtR (Rv1994c) is the biggest hub in the network, followed by ald (Rv2780). Most over-represented pathways among the first neighbors of each of the hubs are mentioned.

Response network analysis has been proven to be an elegant tool in strategic identification of drug targets in pathogens due to its capacity of delineating the most perturbed cellular processes and cross-talk in a condition of interest Padiadpu et al. (2016); Bhosle et al. (2020). Here, we have constructed a knowledge-based functional interactome MtbPPIN and generated dormancy response networks (Dormancy_TPN_) for each of the *in vitro* models and stages (see section 2). The Dormancy_TPN_ for a condition captured the top active and repressed interactions based on a stringent path-cost based filter. This not only included metabolic enzymes but also other important proteins, e.g., transcriptional regulators, structural components etc. In our previous step of the target identification pipeline, 19 upregulated and 165 downregulated reaction-associated genes were shortlisted. Proteins with higher expression or activity level are more viable drug targets since inhibiting those with a chemical or biological compound is more practicable than increasing the activity of any protein with external intervention. Thus, for the next stage of the pipeline, we treated the up and downregulated candidates as two separate groups (Figure 1). First, we shortlisted the proteins captured in the Dormancy_TPN_s of 3 or more *in vitro* dormancy models. 4 of the 19 upregulated candidates were found to pass the response network filter. These were Rv2590, Rv3515c, Rv1832, and Rv2780 (Table 3). These four genes were selected as final shortlisted targets for dormant *Mtb* (Figure 1 and Table 3). Since the Dormancy_TPN_s also provided crucial information on transcriptional regulation of different genes, we aimed to find repressors of the genes associated with downregulated reactions from the previous step. Repressors of such genes could be expected to be upregulated themselves, thereby acting as potential drug targets. With this aim, we first filtered those of the 165 genes that were captured in Dormancy_TPN_s of 3 or more *in vitro* dormancy models, retaining 91. Next, we obtained all known and predicted transcriptional regulators in *Mtb* from the Mycobrower and other resources (Balázsi et al., 2008; Zeng et al., 2012; Galagan et al., 2013; Minch et al., 2015). Edges capturing regulatory interactions were selected from the Dormancy_TPN_s. These interactions were further pruned to retain only the edges occurring in at least 50% of the dormancy conditions, where any of the 91 genes were the targets of the regulators. This exercise provided a list of 25 transcriptional regulators. Two of these regulators were found to be upregulated themselves and connected to genes significantly downregulated in all the dormancy conditions, confirming transcriptional repression (Figure 4). These two transcriptional repressors of metabolic genes, Rv1994c and Rv0324, were added to the final list of shortlisted candidate targets (Figure 1 and Table 3).

**Table 3:** Summarized details of the final shortlisted drug target candidates for dormant *Mtb*.

**Gene ID**	**Gene name**	**Protein name**	**Function**	**Protein length**	**Gene expression status**	**Network properties**
Rv2590	fadD9	Probable fatty-acid-CoA ligase FadD9 (Fatty-acid-CoA synthetase)	Involved in lipid degradation, specific function unknown	1168	Upregulated two-to four-folds in all dormancy models	Neighbors: 4, DEG among neighbors: 1
Rv3515c	fadD19	Long-chain-fatty-acid-CoA/3-oxocholest-4-en-26-oate-CoA ligase	Involved in the activation of long-chain fatty acids as acyl-coA and cholesterol degradation	548	Upregulated two to seven-folds in all dormancy models	Neighbors: 1, DEG among neighbors: 1
Rv1832	gcvB	Probable glycine dehydrogenase (decarboxylating)	Component of glycine cleavage system, binds to the α-amino group of glycine	941	Upregulated 3-8 folds in 3 dormancy models	Neighbors: 18, DEG among neighbors: 9
Rv2780	ald	Alanine dehydrogenase	Catalyzes reversible reductive amination of pyruvate to L-alanine, functions as a homohexamer	371	Upregulated 4- to 40-folds in 3 dormancy models	Neighbors: 105, DEG among neighbors: 39
Rv1994c	cmtR	HTH-type transcriptional regulator (CmtR)	Metal-dependent repression of cmt operon promoter, functions as a homodimer	118	Upregulated 1.5- to 17-folds in all dormancy models	Neighbors: 241, DEG among neighbors: 103
Rv0324	Rv0324	Possible transcriptional regulatory protein (Possibly ArsR-family)	Possible transcriptional regulator, specific function unknown	226	Upregulated 1.5- to 3-folds in 3 dormancy models	Neighbors: 26, DEG among neighbors: 14

The list of 91 downregulated genes associated with repressed metabolic reactions also provide us with insights into potentially poor targets for latent TB. A number of drug target candidates in actively growing *Mtb* have been reported in literature (Jamshidi and Palsson, 2007; Raman et al., 2008; Kaur et al., 2017). From the present analysis, we observe that 27 of those predicted active TB targets are not likely to be effective against dormant TB (Supplementary File 6). We tested if known targets for clinically used first and second-line drugs featured in our lists and found that the model predicts most of the currently used anti-TB drugs to show low efficacy against dormant bacilli (Supplementary Table 1).

#### 3.1.4. Supporting Evidence of Functional Importance of Shortlisted Drug Target Candidates From Experimentally Derived Literature

Multiple studies have used omics-based and individual molecular techniques to study genes and proteins playing crucial roles in *Mtb* in different *in vivo* infection models in cells or animals, and *in vitro* conditions such as stress-inducing conditions or dormancy. We used this vastly available knowledge as a fold of validation for the potential of success of our shortlisted drug candidates against latent tuberculosis. Five of our six shortlisted targets [fadD9 (Rv2590), fadD19 (Rv3515c), ald (Rv2780), cmtR (Rv1994c), Rv0324] have been reported to be transcriptionally upregulated in *Mtb* once they infect macrophages and encounter the intra-phagosomal stress (Schnappinger et al., 2003). Another proteomics quantification study showed that fadD9 (Rv2590), gcvB (Rv1832), and ald (Rv2780) levels significantly increase in *Mtb* in *in vitro* hypoxia-induced dormancy model (Schubert et al., 2015). Upregulation of genes involved in lipid degradation, such as fadD9 (Rv2590) and fadD19 (Rv3515c), in a high lipid environment or hypoxia is intriguing because these conditions closely simulate the environment inside a granuloma (Karakousis et al., 2004; Salina et al., 2009). gcvB (Rv1832) and ald (Rv2780), encoding a glycine cleavage complex component and L-alanine dehydrogenase, respectively, have also been observed to be present in high amount in hypoxia-induced dormancy models at transcript and protein levels (Wayne and Lin, 1982; Betts et al., 2002; Schnappinger et al., 2003; Rustad et al., 2008). Glyoxylate shunt and the associated enzymes are critical to non-replicating *Mtb* inside host cells and its survival in a nutrient-limited condition (McKinney et al., 2000; Puckett et al., 2017). Fatty acid degradation can be directly linked to increased flux through glyoxylate shunt as an alternative source of energy. Increased fatty acid oxidation also increases cellular demand for NAD+ since it is utilized in the process. Higher activity of L-alanine dehydrogenase (ald/Rv2780), can help the bacterial cell to meet this demand of NAD+ in the reversible pyruvate deamination reaction (Schnappinger et al., 2003). It may also support the maintenance of NAD pool in dormant *Mtb* in anaerobic condition when terminal electron acceptor oxygen becomes limiting (Starck et al., 2004; Gopinath et al., 2015). ald (Rv2780) has been suggested as a druggable target for dormant *Mtb* in previous *in silico* analysis as well (Murphy and Brown, 2007; Defelipe et al., 2016). The two transcriptional regulators, cmtR (Rv1994c) and Rv0324, identified as drug target candidates, have also been implicated to be crucial in various dormancy inducing models. cmtR (Rv1994c) is reported to be strongly induced by copper-mediated toxicity in replicating as well as dormant mycobacteria (Salina et al., 2018), whereas Rv0324 plays a major role in *Mtb* enduring hypoxic response and in the tolerance against Bedaquiline (Peterson et al., 2016, 2020). Overall, our shortlisted target candidates have been linked to *Mtb* dormancy is various models of dormancy induction and it provides additional confidence in our prediction of their potential in treating latent tuberculosis.

#### 3.1.5. Pruning the Target Shortlist as Candidates for Lead Identification

With our systems biology pipeline we have identified 6 drug target candidates for latent tuberculosis treatment. Further, we pruned the list to 3 targets most suitable for lead identification with structural modeling and chemoinformatics analysis. cmtR (Rv1994c) and ald (Rv2780) were linked to the highest number of DEGs (103 and 39, respectively) amongst the 6 shortlisted targets in the Dormancy_*TPN*_ subnetwork (Figure 4). As mentioned in the previous section, ald (Rv2780) is reported to be important in dormancy (Wayne and Lin, 1982; Betts et al., 2002; Schnappinger et al., 2003; Rustad et al., 2008). On the other hand, although cmtR (Rv1994c) has been mostly reported in the context of metal toxicity, it is upregulated in all the dormancy conditions studied and is highly connected in the top perturbed network. Also, of the 6 shortlisted targets, experimentally solved structures are available only for cmtR (Rv1994c) and ald (Rv2780) (Banci et al., 2007; Tripathi and Ramachandran, 2008). The third target candidate fadD19 (Rv3515c) was selected based on functional relevance. Its function is well-elucidated, unlike some of the target candidates in our list. It catalyzes the addition of CoA group to long-chain fatty acids, which can get transferred to the polyketide synthases (Trivedi et al., 2004). It is also reported to catalyze thioesterification of C8 alkanoate side chain of cholestenoate, which is an intermediate of cholesterol degradation (Casabon et al., 2014) and is essential for cholesterol degradation in *Mtb* (Griffin et al., 2011). Accumulation of lipids, especially cholesterol, inside host granuloma and its utilization by *Mtb* plays a critical role in the survival and dormancy of the bacteria (VanderVen et al., 2015). All the three selected targets were found to have no close homologue in the human proteome. ald (Rv2780) and fadD19 (Rv3515c) were suggested as druggable targets in the tuberculosis drugome (Kinnings et al., 2010). In summary, cmtR (Rv1994c), ald (Rv2780), and fadD19 (Rv3515c) were selected as the most relevant targets to pursue in the next phase of our study.

### 3.2. Associating Potential Lead Molecules With Drug Target Candidates

**Figure 5:**
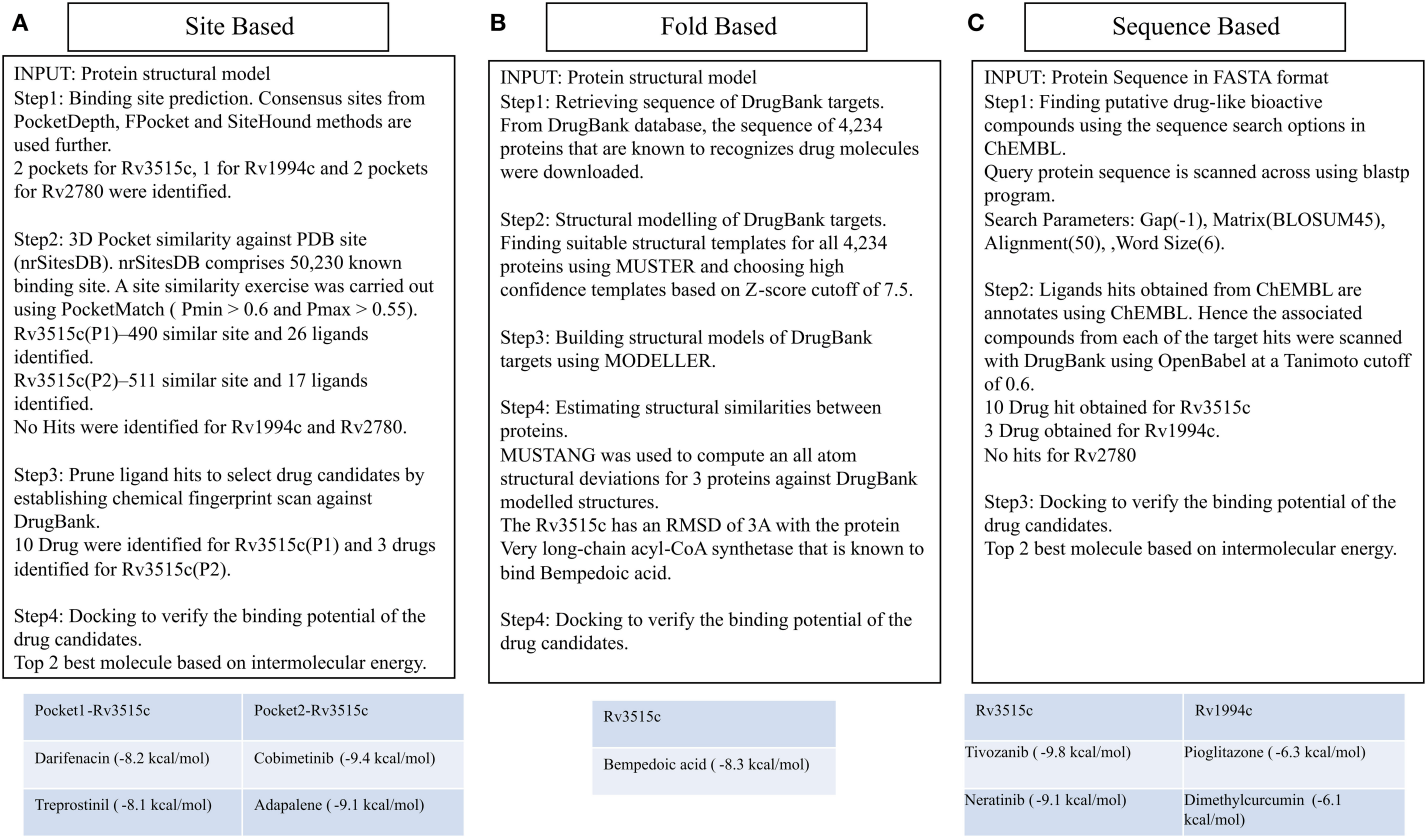
Different ways of associating targets with DrugBank drugs. The site-based association uses binding site similarity as a metric for assigning function to a protein. Consensus pocket prediction exercise yields two pockets for the protein fadD19 (Rv3515c), 1 pocket for cmtR (Rv1994c), and two pockets for ald (Rv2780). Site matching exercise on PDB site library gave 43 ligands that could bind with fadD19. Ligand hits were then pruned by establishing chemical fingerprints on DrugBank molecules. Cobimetinib and darifenacin came out as the best repurposable drug candidate for fadD19. No hits were identified for ald and cmtR. Fold based annotation involves mapping drugs to the targets from the known drug binding proteins. The 3D structure of 4,234 proteins was built using MUSTER and MODELER, of which one hit, very long-chain acyl-CoA synthetase, binding to bempedoic acid share a fold level similarity with fadD19. No hits were identified for ald and cmtR. Finally, sequence derived analysis uses the sequence search option in ChEMBL to identify bioactive compounds. 10 drugs were identified for fadD19, 3 for cmtR. No hit was obtained for ald.

We aimed to identify drug molecules that can be potentially repurposed for latent TB or would provide clues for designing new ligands for the selected targets. We utilized a combined structural modeling and chemoinformatics approach for this purpose as schematically represented in Figure 5.

#### 3.2.1. Structural Characterization of Shortlisted Drug Target Candidates

Three proteins (fadD19, ald, and cmtR) that were identified as target candidates in the steps described earlier were analyzed with a goal of identifying possible lead candidates. Structural insights from these proteins were derived based on available *in silico* structure prediction methods and high-resolution templates available from PDB. MUSTER, a threading algorithm, was used to find the optimal template based on query coverage with the target structure and the Z-score (Wu and Zhang, 2008). With a Z-score threshold greater than 7.5, all three proteins were found to have high confidence templates, which were then modeled using Modeler software (Webb and Sali, 2016). cmtR (Rv1994c) is a winged helical DNA binding transcriptional repressor of 118 residues which senses lead and cadmium. CmtR exists both as monomer and dimer with Cys-102 from one subunit and Cys57, Cys-61 from another subunit forming cadmium binding site. The far UV CD spectra reveal the major portion of this protein is made of α helical segments (Banci et al., 2007).

ald (Rv2780) is an L-Alanine dehydrogenase catalyzing the conversion of ammonia and pyruvate to L-alanine. It constitutes two domains, substrate binding and NAD binding connected by two alpha helices (Jackson et al., 2016). Both, cmtR (Rv1994c) and ald (Rv2780) had a crystal structure already deposited in PDB and hence the same protein was used for structural modeling (Banci et al., 2007; Tripathi and Ramachandran, 2008). For fadD19 (Rv3515c), structural information was not available. Nevertheless, all three proteins have good query coverage and hence considered them for further analysis. Supplementary Table 2 shows the result of structural modeling exercise along with the template taken from MUSTER fold library and the extent of structural deviation in Å.

#### 3.2.2. Characterizing the Drug Binding Nature of Target Candidates

Using the modeled structures as input, the drug binding capabilities of each of them against DrugBank molecules were studied comprehensively via three independent approaches: (i) Site-level study which involves binding site prediction, site similarity against known PDB ligand binding site and ligand similarity between PDB ligands and DrugBank molecules, (ii) Measuring structural similarity against known DrugBank targets by doing a pairwise structural alignment of six proteins onto known drug binding proteins and (iii) By measuring the sequence similarity of the query sequence with known DrugBank target sequences.

**Figure 6:**
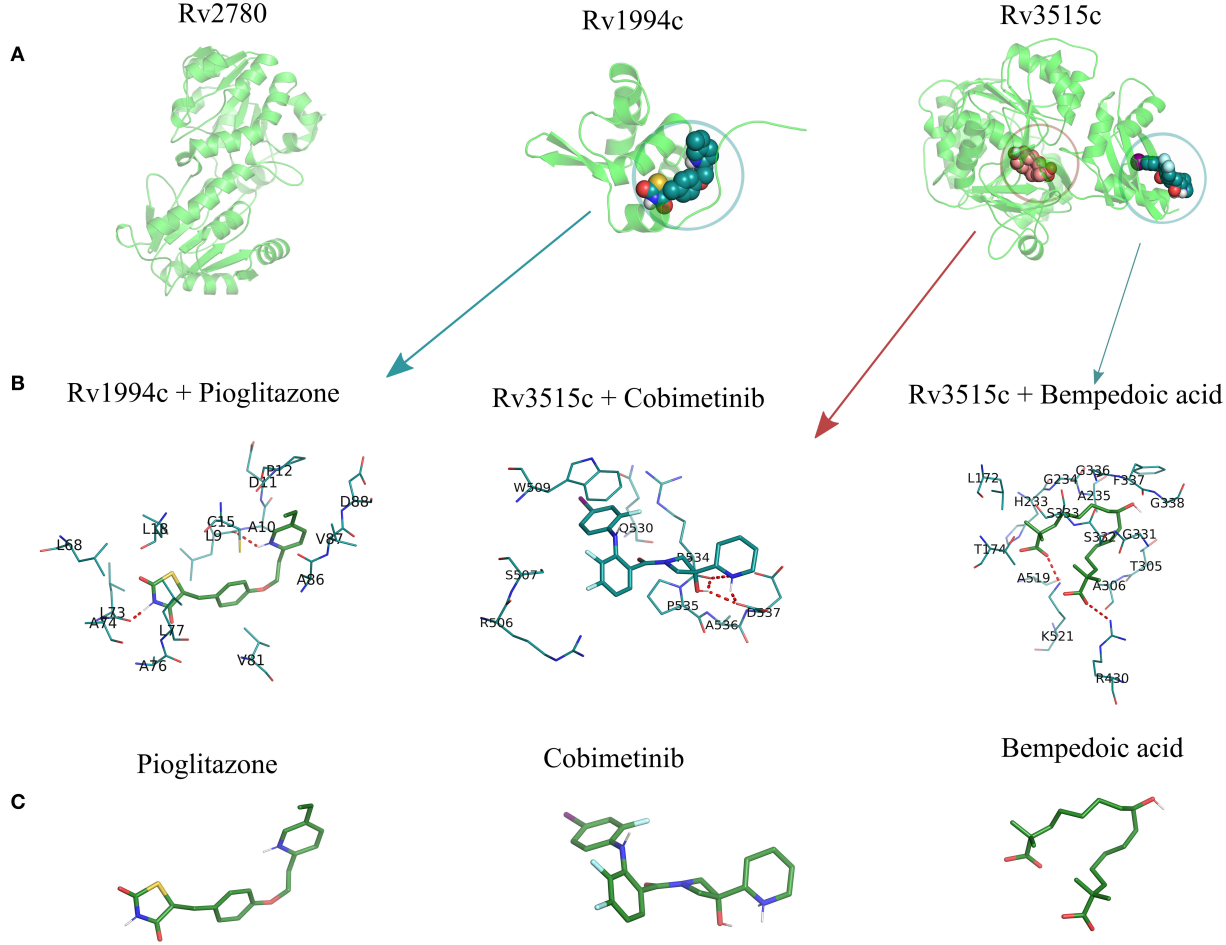
The structural model of the three shortlisted target candidates predicted along with the pocket identified from the consensus approach. **(A)** The structures predicted for each of the 3 proteins, i.e., ald (Rv2780), cmtR (Rv1994c), and fadD19 (Rv3515c), are represented in cartoon format. **(B)** The docking pose of the best drugs for the targets. fadD19 (Rv3515c) is docked against Bempedoic acid and Cobimetinib, while cmtR (Rv1994c) is docked with Pioglitazone. No drug hits are identified for ald (Rv2780). **(C)** Three-dimensional structures of the 3 docked ligands.

(i) **Binding site prediction:** Consensus binding site prediction yields a total of 5 high confidence pockets for three proteins that can recognize small molecules (Figure 6). The nature and type of ligand binding to individual sites could be analyzed based on pre-existing resources. PDB currently holds unique 50,230 structural complexes excluding buffer ligands, thus serving as a knowledge base of protein-ligand interaction information (Rose et al., 2017). A fast site comparison study was used to assign different PDB ligands to consensus pockets using the in-house PocketMatch algorithm (Yeturu and Chandra, 2008). All 5 high confidence pockets were scanned against 50,230 known PDB binding sites in an all-vs-all manner. The output of PocketMatch results was pruned based on two scoring schemes, P_max_ (to encompass global similarities between sites) and P*min* (to capture local similarities between sites). From our previous study, it was shown that P_max_ > 0.5 and P_min_ > 0.6 reflect meaningful similarities between a given pair of sites, hence the same cutoff was employed here (Anand et al., 2012). The binding sites of all three proteins have similarities with the sites of proteins recognizing natural ligands. For example sites of ald (Rv2780) were predominantly mapped to NAD and FAD liganded sites. Associations made with natural ligand sites were not considered as we were only interested in finding candidate drugs. To accomplish that, we used open-babel and compared the 149 ligand hits obtained from PocketMatch against ligands deposited in DrugBank. DrugBank stores ligand molecules based on one of these groups, Approved, Nutraceutical and Investigational and we screened only those in the Approved category as most of the commercial drugs belong to this class. Of 11,414 entries being deposited in DrugBank, 10 drugs were identified as hits for fadD19 (Rv3515c) at a given Tanimoto cutoff of 0.70 (Table 4 and Supplementary Figure 4). No hits were obtained for cmtR (Rv1994c) and ald (Rv2780). For fadD19 (Rv3515c), the binding energy of each of the drugs was calculated using Autodock. Full results describing the name of each drug along with the predicted binding affinity with the respective target is provided in Supplementary File 7.

**Table 4:** A list of top drug hits identified from three distinct methods.

**Target genes**	**DrugBank ID**	**Drug name**	**Known drug action**	**Methods employed**	**Calculated binding energy (kcal/mol)**
**Rv3515c (fadD19)**	**DB05239**	**Cobimetinib**	**To treat metastatic melanoma. Inhibits protein MAP2K1**	**Site level**	−10.09
Rv3515c (fadD19)	DB00210	Adapalene	To treat acne vulgaris	Site level	−9.85
Rv3515c (fadD19)	DB00496	Darifenacin	Treats overactive bladder by blocking M3 muscarinic acetylcholine receptors	Site level	−9.30
Rv3515c (fadD19)	DB00605	Sulindac	It is a nonsteroidal anti-inflammatory drug	Site level	−8.80
Rv3515c (fadD19)	DB00563	Methotrexate	An immunosuppressant, inhibit enzymes responsible for nucleotide synthesis	Site level	−8.74
**Rv3515c (fadD19)**	**DB11936**	**Bempedoic acid**	**An ACL inhibitor used in reducing LDL cholesterol levels**	**Structure based**	−6.51
Rv3515c (fadD19)	DB11800	Tivozanib	Inhibitor of vascular endothelial growth factor receptor	Sequence based	−9.85
Rv3515c (fadD19)	DB11828	Neratinib	An irreversible tyrosine kinase inhibitor used in treatment of breast cancer	Sequence based	−9.16
**Rv1994c (cmtR)**	**DB01132**	**Pioglitazone**	**Uses to treat type 2 Diabetes mellitus**	**Sequence based**	−6.29
Rv1994c (cmtR)	DB06133	Dimethylcurcumin	An anti androgen that enhances degradation of androgen receptors.	Sequence based	−6.16
Rv1994c (cmtR)	DB00412	Rosiglitazone	An antidiabetic drug works by activating peroxisome proliferator-activated receptors	Sequence based	−5.75

*For each identified DrugBank hit, the common name and the known mechanism of drug action is provided along with the theoretical binding energy calculated using AutoDock*.

(ii) **Prediction of drug binding from structures:** Next we mapped drugs to our identified targets based on the existing structural data available in databases such as DrugBank. For all drug molecules available in DrugBank, the UniProt IDs of the proteins that were known to recognize them were retrieved. The sequence information of these proteins was then fetched from the UniProt website amounting to a total of 4,555 sequences. A structural library for DrugBank targets was created for all 4,555 proteins using the MUSTER programme. High confidence templates were taken based on reported Z-score cutoff of 7.5 resulting in a total of 4,234 proteins that are suitable for structural modeling. The final structure was built using MODELER. To transfer functional annotation from one structure to another, we employed a protein structure alignment approach to computing the extent of deviations between structures using a well-known structure alignment software called MUSTANG (Konagurthu et al., 2006). All 3 proteins were aligned onto 4,234 structures in a pairwise manner resulting in 12,702 combinations, out of which only 1 pair aligned with an RMSD less than 3Å. The protein Very long-chain acyl-CoA synthetase (UniProt ID: O14975) was found to be structurally similar to fadD19 (Rv3515c) and was known to bind Bempedoic acid, a drug for the treatment of hypercholesterolemia. As both fadD19 (Rv3515c) and O14975 possess global fold level similarity, we believe Bempedoic acid can be used as a repurposable drug candidate.

(iii) **Deriving drug binding from sequence analysis:** We explored associating drugs to our targets from proteins that are known to bind drug molecules. Sequence-based search option in ChEMBL was used to get the list of related proteins along with their ChEMBL compounds in 2D sdf format. Conversion from 2D to 3D coordinates was made using Open Babel software (O'Boyle et al., 2011). Many of the compounds available in ChEMBL are not annotated and are completely new, but we were only interested in drugs that were approved. To do this, the structure of all ChEMBL molecules was scanned against approved drugs in DrugBank. At a Tanimoto cutoff of 0.70, we obtained 14 drug molecules for fadD19 (Rv3515c) and 3 for cmtR (Rv1994c) (Table 4, Supplementary Figure 4, and Supplementary File 7). No drug hits were obtained for ald (Rv2780).

## 4. Discussion

Large-scale systems-level modeling approaches are becoming increasingly popular to understand biological systems due to its capacity of providing insights into their complex molecular networks, pathways and crosstalk. High-throughput methods are of particular importance in the field of drug discovery due to the urgent need for new and improved drugs for most of the diseases. Although chemical screening methods have been successfully utilized for drug identification for many diseases (Ananthan et al., 2009; Siqueira-Neto et al., 2010; Debnath et al., 2012), the large scale experiments incur a high cost and time, and in many cases, the mechanisms of action of the drugs remain a black-box. Systems-level modeling provides an efficient top-down approach for predicting potential drug targets in biological systems based on functionality and to associate lead molecules with them.

Latent TB is a widespread disease and is difficult to tackle due to our limited understanding of the condition. Majority of the anti-TB drugs target cellular processes critical to replicating pathogens (Campbell et al., 2001; Timmins and Deretic, 2006; Nagabushan and Roopadevi, 2014). Two of the most well-established phenomena associated with latent tuberculosis is the non-replicative state of the bacteria and its significant metabolic rewiring (McKinney et al., 2000; Betts et al., 2002; Voskuil et al., 2004; Gengenbacher et al., 2010). Therefore an understanding of the differences in the metabolic processes and their regulations between actively growing *Mtb* and dormant ones holds the potential of providing critical clues for tackling latent TB. In this study, we have utilized a genome-scale metabolic model of *Mtb* (iEK1011) integrated with transcriptome information to identify the key differences between the replicating and dormant pathogen. One major limitation in studying latent TB remains the lack of a model system that correctly simulates the conditions of the dormant bacteria inside human granuloma. Different *in vitro* models have been established to achieve the dormant state of *Mtb* but each of these has been limited in the use of any one or few of the stress conditions that the bacteria face inside the host, thereby mimicking the *in vivo* condition only partially. In order to overcome this limitation, we have considered different models of dormancy and identified the metabolic alterations that occur across different stress conditions. Such alterations are most likely to be occurring in dormant *Mtb* inside the host and be critical for their survival, making the associated enzymes interesting targets for drug discovery. We further filtered the targets using transcriptome integrated genome-wide protein-protein interaction network (response network) analysis. This step provided a broader perspective into the perturbations occurring in dormant *Mtb* as it considers not only the metabolic network but all the other molecular and functional interactions as well. The metabolic enzymes captured in the top-perturbed response networks for dormancy along with being associated with reactions with the highest flux changes across dormancy conditions could be considered as high confidence drug targets. fadD9 (Rv2590), fadD19 (Rv3515c), gcvB (Rv1832), and ald (Rv2780) were shortlisted from this pipeline as drug target candidates. Since overexpressed proteins are easier to inhibit with a drug than targeting any gene or protein that is downregulated, we intended to focus on upregulated proteins as drug target candidates. Predictably, most of the metabolic reactions were downregulated in dormant *Mtb*. This led us to use the regulatory interactions from dormancy response networks to find transcriptional regulators that might be associated with such downregulated metabolic reactions. We found two transcriptional regulators, cmtR (Rv1994c) and Rv0324, to be regulating multiple metabolic reactions while being upregulated themselves. All of these 6 target candidates were closely connected with each other in the response network, suggesting a functional interaction between them. The metabolic enzymes in the shortlist are involved in lipid metabolism and the glyoxylate cycle. These processes are critical in the maintenance of dormancy in Mtb and survival inside nutrient-limited host granuloma environment (McKinney et al., 2000; VanderVen et al., 2015; Puckett et al., 2017), showing that the functional relevance of the identified drug target candidates.

In the next step we further pruned the target shortlist to 3 candidates, fadD19 (Rv3515c), cmtR (Rv1994c), and ald (Rv2780), based on their biological significance and network properties, for lead association. The structures of the target proteins were obtained from experimentally solved structures or were generated with homology modeling. To identify potential lead molecule associations three independent approaches were taken. In the first approach, we predicted the binding sites for each of the target candidates using a consensus between three orthogonal methods, PocketDepth, SiteHound, and FPocket (Kalidas and Chandra, 2008; Ghersi and Sanchez, 2009; Le Guilloux et al., 2009). Each of these methods has its own strengths and limitations and employs a different strategy for binding site identification and deploying a consensus approach provides higher confidence in the predicted binding sites. Multiple resources were then scanned to find ligands and drugs that can bind to the high-confidence binding sites in the target proteins. In the second approach, structural information of known drug-target interactions from DrugBank was used to associate drugs with our three candidate proteins by finding similarity between structural folds. In the third approach, lead-target associations were obtained using a sequence level similarity analysis in the ChEMBL database. The utilization of three different approaches enables us to derive the strengths of each of the methods and explore the available resources to the fullest. We have successfully associated multiple known natural ligands such as NAD and FAD as well as 28 drug molecules with our shortlisted targets. These associated drug leads are already used as approved drugs for other disease and could therefore be readily explored for the possibility of repurposing.

In summary, this study provides a ready shortlist of potential drug targets for latent tuberculosis to be further studied. Multiple known drug molecules have been associated with the shortlisted targets which can either be explored as possible repurposable drugs or at the very least will provide significant clues for chemically designing new drug molecules for the target proteins.

## 5. Equations

For active response network,

(1) $$\begin{array}{r} NW_{i}=FC\left(i\right)=\frac{\left(Expression\;of\;gene\;‘i’\;in\;dormancy\right)}{\left(Expression\;of\;gene\;‘i’\;exponential\;growth\right)} \end{array}$$

For repressed response network,

(2) $$\begin{array}{r} NW_{i}=FC\left(i\right)=\frac{\left(Expression\;of\;gene\;‘i’\;exponential\;growth\right)}{\left(Expression\;of\;gene\;‘i’\;in\;dormancy\right)} \end{array}$$

Where NW means node weight, FC stands for fold change in gene expression and ‘i' indicates the gene.

The edge weight (EW) between nodes ‘i' and ‘j' is calculated based on the node weights as,

(3) $$\begin{array}{r} EW_{ij}=\frac{1}{\sqrt{NW_{i}\times NW_{j}}} \end{array}$$

(4) $$\begin{array}{r} Path\;Cost=\frac{\sum_{i=1}^{n}EW_{i}}{Number\;of\;nodes\;in\;path} \end{array}$$

## Data Availability Statement

Publicly available datasets were analyzed in this study. This data can be found here: GEO repository (https://www.ncbi.nlm.nih.gov/geo/) under IDs GSE8786, GSE10391, GSE66408, and GSE84554.

## Author Contributions

NC conceptualized, designed, and supervised the study. UB performed the data curation and analysis and interpretation of target identification. SS performed the data curation and analysis and interpretation of lead identification. AS provided critical insights and discussions. UB and SS wrote the first draft of the manuscript. NC revised the manuscript. All authors read and approved the submitted version.

## Conflict of Interest

NC is associated with qBiome Research Pvt Ltd and HealthSeq Precision Medicine Pvt Ltd, it has no role in this manuscript. The remaining authors declare that the research was conducted in the absence of any commercial or financial relationships that could be construed as a potential conflict of interest.

## Abbreviations

FAD: Flavin Adenine Dinucleotide
FBA: Flux Balance Analysis
GEM: Genome-scale Metabolic Model
GEO: Gene Expression Omnibus
GPR: Gene Protein Rule
MDR: Multidrug Resistant
NAD: Nicotinamide Adenine Dinucleotide
PDB: Protein Data Bank
TB: Tuberculosis
XDR: Extremely Drug Resistant.
