# Supplementary material for: A Multi-Pronged Computational Pipeline for Prioritizing Drug Target Strategies for Latent Tuberculosis
Source: Front Chem. 2020 Dec 14;8:593497. doi: 10.3389/fchem.2020.593497 (PMC7767875; doi:10.3389/fchem.2020.593497)
Supplement: Supplementary file 1 [file Data_Sheet_1.PDF]

A Multi-pronged Computational Pipeline for Prioritizing Drug Target Strategies for Latent Tuberculosis

Ushashi Banerjee [1,†], Santhosh Sankar [1,†], Amit Singh [2] and Nagasuma Chandra [1,3,*]

[1] *Department of Biochemistry, Indian Institute of Science, Bangalore, India*
[2] *Centre for Infectious Disease Research, Indian Institute of Science, Bangalore, India*
[3] *Center for Biosystems Science and Engineering, Indian Institute of Science, Bangalore, India*
[†] *Equal Contribution*

Correspondence*:
Nagasuma Chandra
nchandra@iisc.ac.in

1 SUPPLEMENTARY DATA

Supplementary Figures

1.1 Supplementary Tables

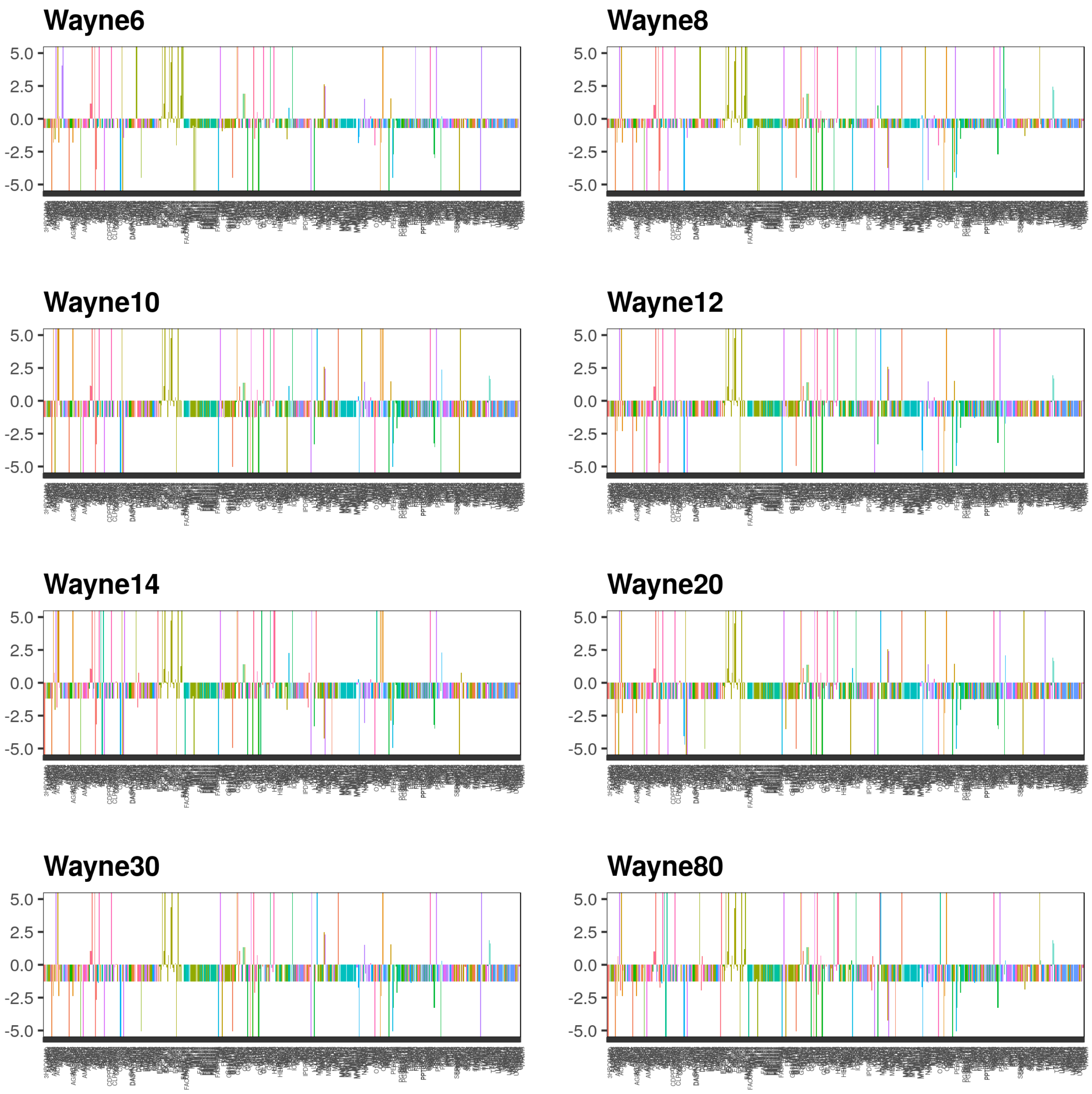

Figure 1. Flux fold change of all non-zero reactions in Hypoxia model of dormancy at different days after stress induction in comparison to the exponential growth phase. X axis in each contains the reactions with non-zero fluxes and Y axis shows the fold change value. Most of the reactions are downregulated during dormancy

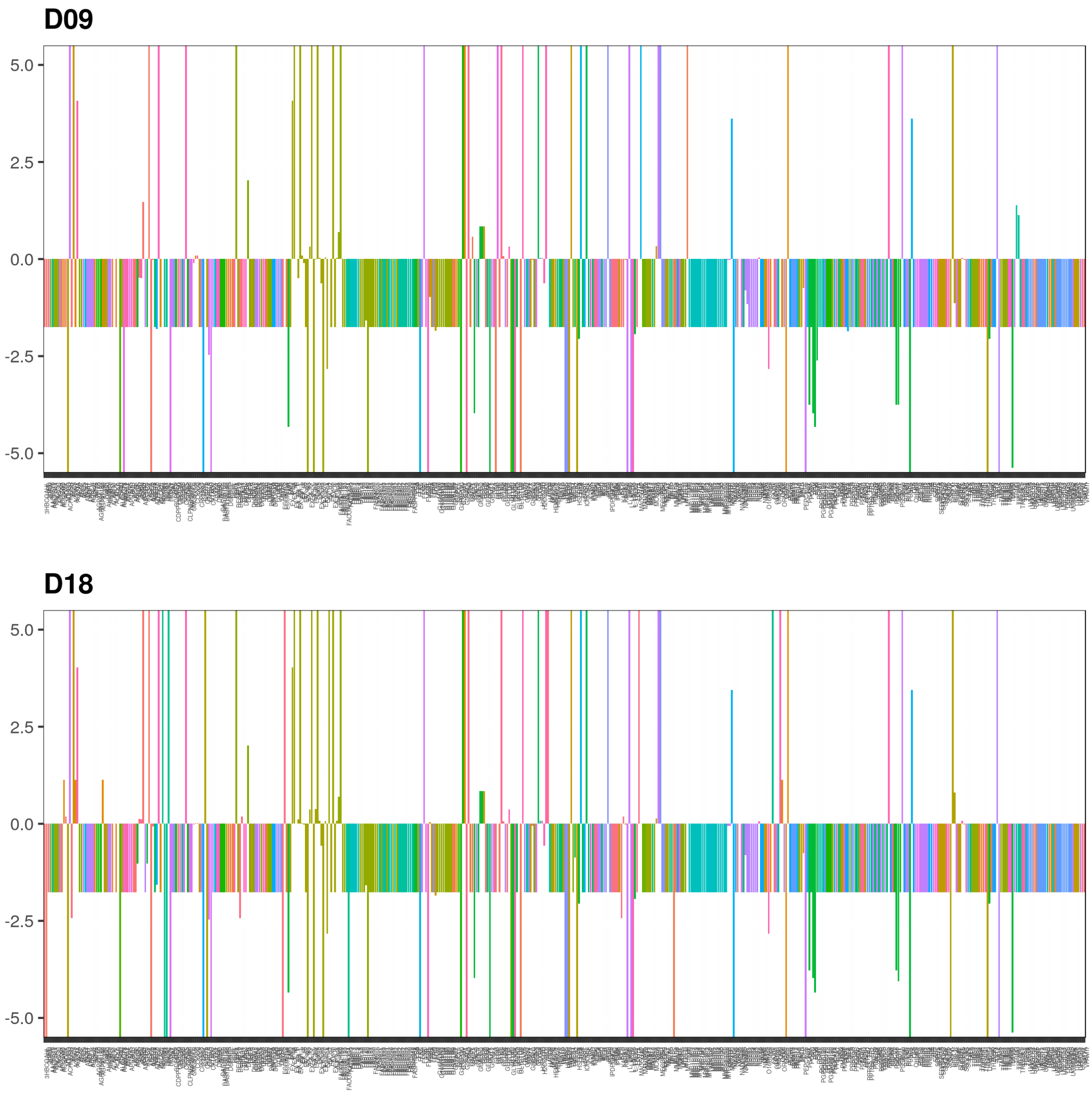

Figure 2. Flux fold change of all non-zero reactions in multiple model of dormancy at different stage after stress induction in comparison to the exponential growth phase. X axis in each contains the reactions with non-zero fluxes and Y axis shows the fold change value. Most of the reactions are downregulated during dormancy

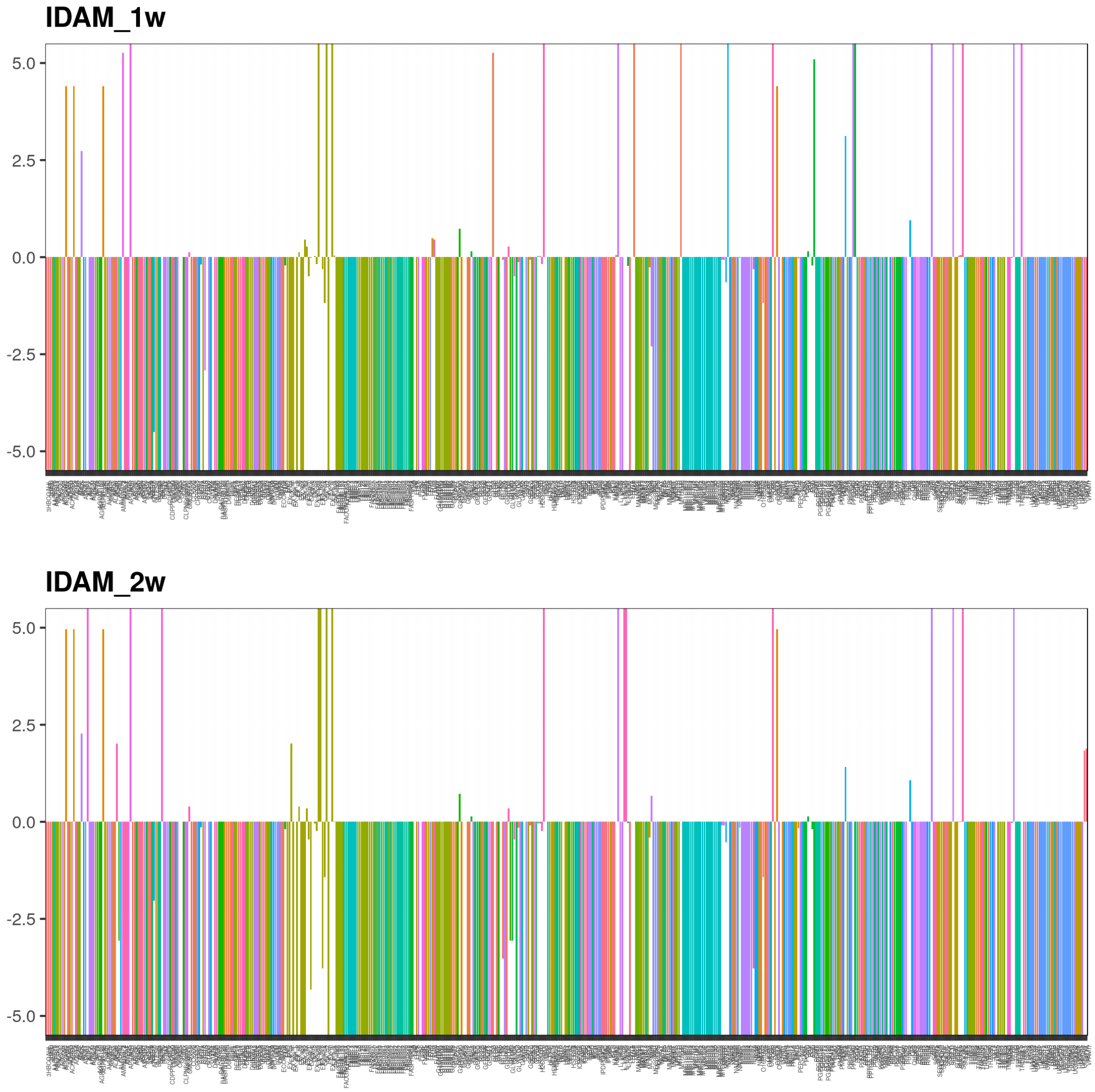

Figure 3. Flux fold change of all non-zero reactions in Iron restriction model of dormancy at different stages of dormancy in comparison to the exponential growth phase. X axis in each contains the reactions with non-zero fluxes and Y axis shows the fold change value. Most of the reactions are downregulated during dormancy

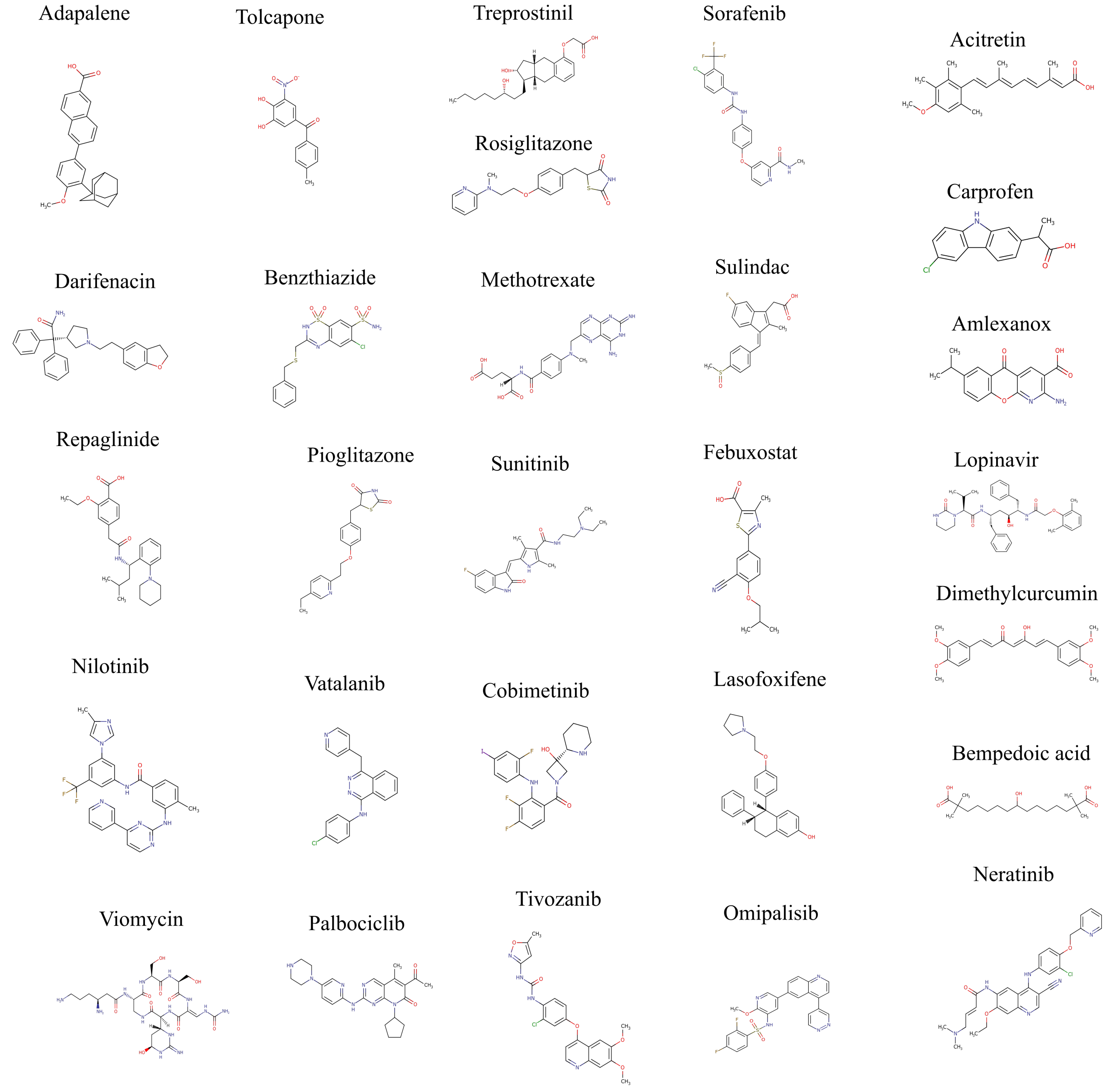

Figure 4. 2D structure of 28 repurposable drugs that are identified from this work.

Drug Name	Target Gene	Is it a Metabolic Gene	Flux in Hypoxia	Flux in Multiple Stress	Flux in K+ Deficiency	Flux in Iron Deficiency	Gene Expression Status in ≥3 dormancy	Predicted Efficacy
Isoniazid	inhA (Rv1484)	Yes	Repressed	Repressed	Repressed	Repressed	Not DEG	Low
Rifampicin	rpoB (Rv0667)	No	-	-	-	-	Up in Hypoxia and K+ Deficiency, Down in Multiple Stress and Iron Deficiency	Cannot Comment
Ethambutol	embABC (Rv3794, Rv3795, Rv3793)	Yes	Repressed	Repressed	Repressed	Repressed	Downregulated	Low
Bedaquiline	atpE (Rv1305)	Yes	Repressed	Repressed	Repressed	Repressed	Downregulated	Low
Delamanid	fbiABC (Rv3261, Rv3262, Rv1173)	Yes	Zero Flux	Zero Flux	Zero Flux	Zero Flux	Downregulated	Low
Moxifloxacin	gyrA (Rv0005), gyrB (Rv0006)	No	-	-	-	-	Downregulated	Cannot Comment
Cycloserine	ddl (Rv2981c)	Yes	Repressed	Repressed	Repressed	Repressed	Not DEG	Low
Streptomycin	rpsL (Rv0682)	No	-	-	-	-	Not DEG	Cannot Comment

Table 1. Prediction of the efficacy of commonly used first and second line anti-TB drugs against latent TB using our approach. Common drugs where the target gene is not well elucidated are not included in this list.

Sl. No.	Gene Name	Template Fold used from MUSTER	Query Coverage in %	Sequence Identity	Z-score
1	Rv1994c	2jscB	82.2	100	17.7
2	Rv2780	2vhxE	100	100	20.78
3	Rv3515c	4wv3A	92	23.8	22.59

Table 2. The summary of the main results obtained from structural modelling exercise. The sequence identity of two query proteins (Rv1994c and Rv2780) with their target is 100% indicating the query and target are the same. The sequence identity of Rv3515c is low, but the query coverage and structural alignment were good. All three proteins have very good structural alignment with their template and hence all of them were taken further
